# Clinical study of single nucleotide polymorphism-based chromosome microarray analysis in the etiological diagnosis of fetal congenital heart disease

**DOI:** 10.1038/s41598-025-23787-1

**Published:** 2025-11-14

**Authors:** Yinxia Zheng, Shuyuan Xue, Guilan Ding, Luhan Zhang, Guifeng Ding

**Affiliations:** 1 School of Public Health, Xinjiang Medical University, Urumqi, Xinjiang China; 2 Urumqi Maternal and Child Health Hospital, Urumqi, Xinjiang China; 3 Xinjiang Clinical Research Center for Perinatal Diseases, Urumqi Maternal and Child Health Hospital, Urumqi, Xinjiang China

**Keywords:** Pregnant women, Fetus, Congenital heart disease, Single nucleotide polymorphism, Chromosome microarray analysis, Cardiology, Health care, Molecular medicine

## Abstract

This study investigates the clinical application of single-nucleotide polymorphisms (SNP)-based chromosome microarray analysis (CMA) in the etiological diagnosis of fetal congenital heart disease (CHD). We 5,116 amniotic fluid samples collected through amniocentesis from January 2022 to December 2024 in Urumqi, Xinjiang, China. Based on fetal ultrasound findings, structural abnormalities, and specific types, CHD was categorized into four groups: isolated CHD, non-isolated CHD, non-CHD, and a normal group. The incidence of aneuploidies were highest in non-isolated CHD cases (16.91%), approximately five times higher than that in cases with isolated CHD (3.8%) (*P* < 0.001). The incidence of pathogenic copy number variants (CNVs) were similar across groups (2.11%–3.68%). The non-isolated CHD group demonstrated a significantly higher incidence of trisomy 21 (8.82%) and trisomy 18 (5.88%) compared to other groups (*P* < 0.001). Among the pathogenic CNVs, we identified five cases of deletions (22q11.2) in the isolated CHD group, eight losses (15q11.2),and 11 losses (22q11.2) in the normal group. SNP-based CMA enhances the detection of abnormal CNVs in fetuses with CHD, offering medical reference for diagnosing chromosomal etiologies and enabling precise genetic counseling. For clinical practice, SNP-based CMA should be strongly recommended for non-isolated CHD fetuses, and considered as a supplementary test for isolated CHD fetuses.

**Supplementary Information:**

The online version contains supplementary material available at 10.1038/s41598-025-23787-1.

## Introduction

Congenital heart disease (CHD) encompasses diseases affecting heart morphology and function due to abnormalities in the development of heart, valve, or vascular tissue structures. CHD is mainly divided into ventricular septal defect (VSD), atrial septal defect, patent ductus arteriosus, tetralogy of Fallot (TOF), and over two dozen additional subtypes^1^. CHD hinders normal fetal development, leading to abortion, stillbirth, and other adverse pregnancy outcomes. Although over 90% of fetal CHD patients survive to adulthood^2^, approximately 13% of cases are associated with structural or functional defects that may lead to neurodevelopmental delays in childhood, thereby seriously affecting the quality of life of children.

Globally, CHD affects approximately 1.35 million infants, with an incidence of 9.1 per 1000 live births^3^. There exist substantial regional variations in the incidence of CHD, with rates of 2.3 per 1000 in Africa, 8.2 per 1000 in Europe, and the highest rate (9.3 per 1000) in Asia^4^.CHD represents the congenital defect with the highest morbidity and mortality in infants worldwide^5^, accounting for 20%–30% of infant deaths and 20% of stillbirths^6^. CHD is the leading cause of death in infants aged 0–5 years, accounting for nearly one-third of all major congenital abnormalities, making it the most prevalent birth defect worldwide^7^. Moreover, CHD imposes substantial burdens on healthcare resources, the quality of population health, and sustainable socio-economic development.

Recent advancements in artificial intelligence and machine learning have shown promise in enhancing the medical diagnosis of fetal heart development abnormalities^8^. However, traditional techniques such as echocardiography and magnetic resonance imaging remain the primary tools for prenatal screening and diagnosis of fetal heart abnormalities. Specifically, echocardiography is highly effective in detecting structural and functional abnormalities in the fetal heart, although it often fails to identify the underlying genetic causes. Regarding genetic testing approaches, traditional karyotyping can detect chromosomal number abnormalities and large-segment structural abnormalities, but it cannot identify small chromosomal copy number variations (CNVs).

Chromosome microarray analysis (CMA) offers a resolution of 50 kb/25 marker losses and 100 kb/25 marker gains, significantly surpassing the resolution of conventional karyotyping, which is approximately 5 Mb. Studies have shown that applying SNP-based CMA to detect fetal specimens with abnormal ultrasound findings can detect an additional 5.4% of abnormal CHD cases compared to karyotype analysis^9^. Approximately 10%–15% of CHD cases are associated with definite genetic syndromes (e.g., Down syndrome). In addition, the incidence of aneuploidy, overall chromosomal abnormalities, and trisomy 18 is significantly higher in CHD than in non-CHD cases^10^.

SNP-based CMA is an emerging molecular genetics technology that uses SNP probes to infer CNVs. Through hybridization, laser scanning, and software analysis, this method offers several advantages, including high resolution, throughput, and accuracy^11^.

SNP-based CMA has achieved significant advances in the diagnosis of genetic diseases and tumor genetics research. For example, Jasmine et al.^12^ applied CMA-SNP technology to analyze products of conception from patients with recurrent pregnancy loss (RPL), successfully identifying underlying genetic causes of RPL in these cases. Furthermore, a study of 21 fetuses with 15q11.2 microdeletion demonstrated that over half of the cases had no abnormalities on prenatal ultrasound. Cases with ultrasound features mainly showed isolated malformations, such as an elevated cervical transparent layer, CHD, and structural brain abnormalities. Postpartum patients with 15q11.2 microdeletions are at increased risk for schizophrenia, epilepsy, and other neurological and psychiatric disorders associated with these microdeletions^13^.

However, most studies on SNP-based CMA concerning fetal heart development abnormalities have largely focused on a limited number of specific genes and chromosomal regions—specifically genes associated with common congenital heart defects (e.g., TBX5, GATA4) and chromosomal segments frequently linked to cardiac malformations. Consequently, these studies lack comprehensive genome-wide analysis, failing to explore potential pathogenic variations in less characterized genes or non-coding regions that may equally contribute to fetal cardiovascular abnormalities^14^.

This study aimed to perform genome scans of fetuses with cardiac abnormalities detected on ultrasound to unravel the underlying genetic etiology and pathogenesis. Through thorough analyses of the sites of pathogenic genes and genetic variations, we sought to provide a theoretical basis for early diagnosis, genetic counseling, and personalized treatment of fetal heart abnormalities, as well as introduce new perspectives and methods for studying the pathogenesis of CHD.

## Methods

### Study subjects

This study collected 5,116 amniotic fluid (AF) samples via amniocentesis in Urumqi, Xinjiang, China, from January 2022 to December 2024. Based on fetal ultrasound abnormalities, 2,005 (39.19%) samples exhibited structural abnormalities, while 3,111 (60.81%) cases were classified as normal. In the group with ultrasound-detected structural abnormalities, 373 (7.29%) cases had CHD, and 1,632 (31.9%) cases exhibited non-CHD findings or phenotypes (these included other fetal structural abnormalities such as cleft lip and palate, lateral ventricular widening, renal deficiency, but no fetal cardiac abnormalities; hereafter referred to as non-CHD).

**Fig. 1:**
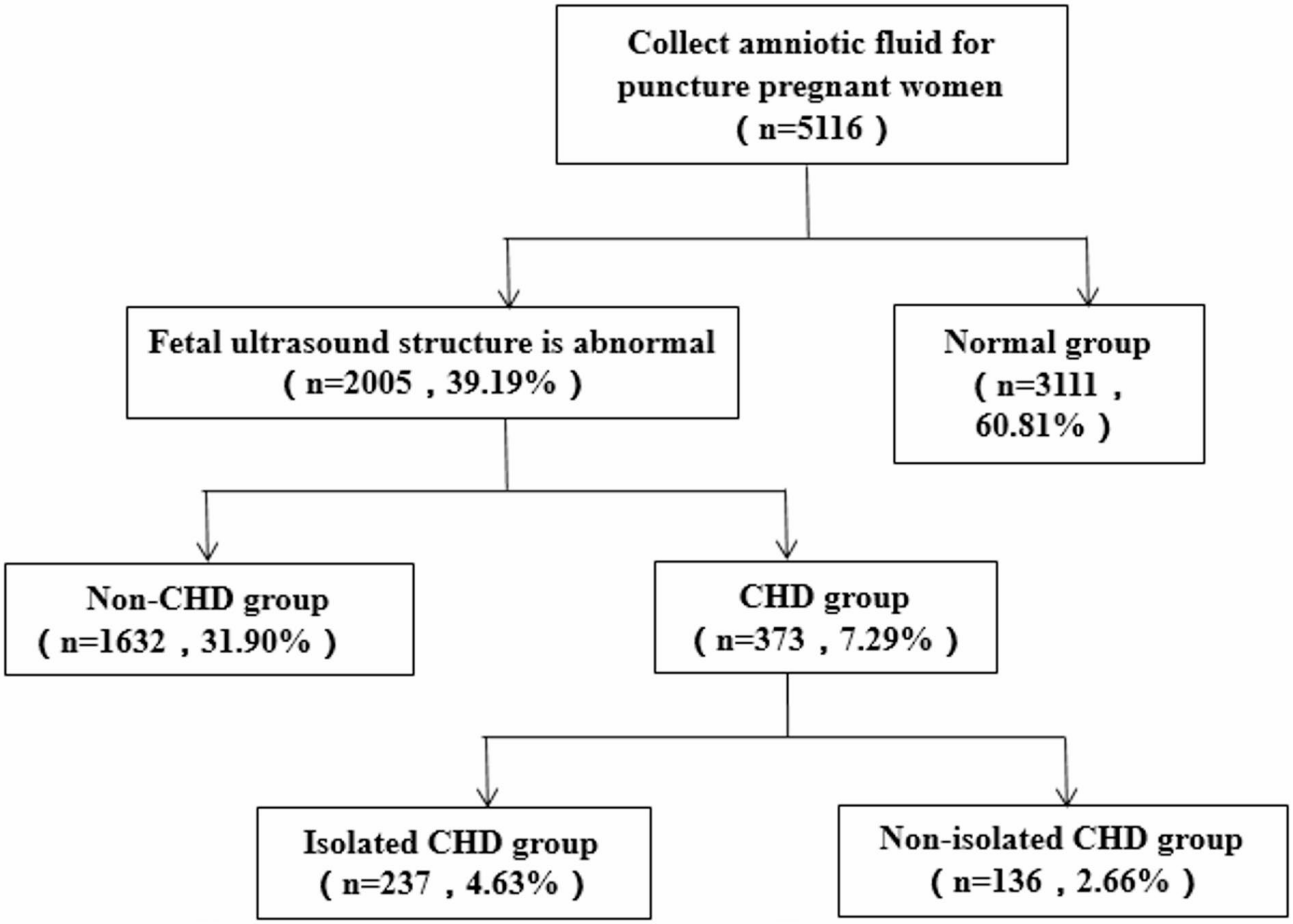
Flow chart of patient cohort inclusion.

In the CHD group, 237 (4.63%) cases had isolated CHD (fetal cardiac abnormalities only), while 136 (2.66%) had non-isolated CHD (cardiac abnormalities accompanied by additional structural abnormalities) (Fig. 1). SNP-based CMA was performed for each group, followed by a retrospective analysis.

The inclusion criteria were as follows: (1) diagnosis of fetal disease by two qualified physicians based on the guidelines of the International Society of Obstetrics and Gynecology Ultrasound^15^. Fetal heart defects were classified according to the International Pediatric and Congenital Heart Disease Code and the International Classification of Diseases version 11 (ICD-11)^16^, which includes isolated and non-isolated CHD; (2) interventional prenatal diagnosis with consent obtained through prenatal genetic counseling; maternal serum biochemical screening (for instance, the levels of PAPP-A and β-HCG in the early stages of pregnancy, AFP, β-HCG, and uE3 in the second trimester, etc.) or non-invasive prenatal testing results that exceed the normal range; adverse pregnancy history (e.g., miscarriage, stillbirth, or delivery of infants with congenital anomalies); maternal age of 35 years or older at conception; oligohydramnios; exposure to teratogenic substances during the first trimester of pregnancy; and maternal mental disorders; and (4) voluntary SNP-based CMA testing.

The exclusion criteria were as follows: (1) consanguineous marriages; (2) pregnant women who received allogeneic blood transfusions, transplantations, or immunotherapies within the last year; (3) those with contraindications to interventional prenatal diagnosis, such as threatened abortion, fever, bleeding tendency, active periods of chronic pathogen infection, and Rh-negative blood type in pregnant women; and (4) incomplete clinical data.

### Ultrasound examination

Screening for fetal neck translucency was conducted between 11 and 13 weeks + 6 days and structural screening was carried out at 20–24 weeks. Complete 2D probes were used, including C2–9 (frequency of 29 MHz) and C1–5 (frequency of 15 MHz), along with fetal heart mode. At least two sonographers qualified in prenatal diagnosis were recruited for the evaluation.

### Collection of amniotic fluid specimens

We extracted 30 mL of AF under ultrasound-guided amniocentesis. Of this, 20 mL was used for cell culture chromosome G band karyotyping, and 10 mL was allocated for CMA detection. The procedure was conducted by a professional physician specializing in prenatal diagnosis, holding an associate senior title or higher.

### Karyotype analysis of chromosome G bands

The collected 20 mL of AF specimens were cultured following standard cytogenetic protocols. Cultured amniotic cells were analyzed using Giemsa staining, followed by karyotyping for all AF samples.

### SNP detection

For all AF samples, 10 mL were tested using a CytoSan 750 K array microarray chip from Affymetrix. DNA was extracted from the amniotic fluid, and its quality and concentration were evaluated. The extracted DNA samples were fluorescently labeled to facilitate hybridization of the sites on the CMA chip for 16–18 h. Following this, signal intensity and fluorescence intensity data were obtained for each site, allowing for genotyping and CNV were performed analysis. The results were queried using the online Human Mendelian Genetic Data System (Online Mendelian Inheritance in Man, OMIM), the Genome variant database (Database of Genomic Variants, DGV), the International Human Cytogenetic Nomenclature System (International System for Human Cytogenetic Nomenclature, ISCN), and other relevant databases and literature. Based on clinical significance, detected genetic variants can be categorized as pathogenic (P), likely pathogenic (LP), and fragments of uncertain significance (variation unknown significance, VUS). All abnormal results were reviewed by at least two senior analysts in the prenatal diagnostic laboratory.

### Statistical analysis

Data were analyzed using SPSS version 26.0 (IBM Corporation, Armonk, NY, USA), and R version 4.3.1 (R Foundation for Statistical Computing, Vienna, Austria) was used for data processing. Measurement data (e.g., age and pregnancy) were described as mean ± standard deviation. Analysis of variance was used when variances were homogenous across groups; otherwise, the rank sum test was used. Count data (e.g., adverse pregnancy history and abnormal outcome typology) were described using frequency or percentage and compared using the chi-square test or Fisher exact probability test. A p-value of < 0.05 was considered statistically significant.

### Ethics statement

All pregnant women provided written informed consent for the amniocentesis and subsequent genetic testing (i.e., SNP-based CMA) involved in this study. This study was approved by the Ethics Committee of Urumqi Maternal and Child Health Hospital (Approval No.: XJFYLL2023061). All methods were performed in accordance with relevant national and international guidelines and regulations, including the *Global Code of Conduct for Research in Resource-Poor Settings* (Council on Health Research for Development, 2017) and the *Declaration of Helsinki* (World Medical Association, 2022).

## Results

### Sociodemographic characteristics of the study subjects

**Fig. 2:**
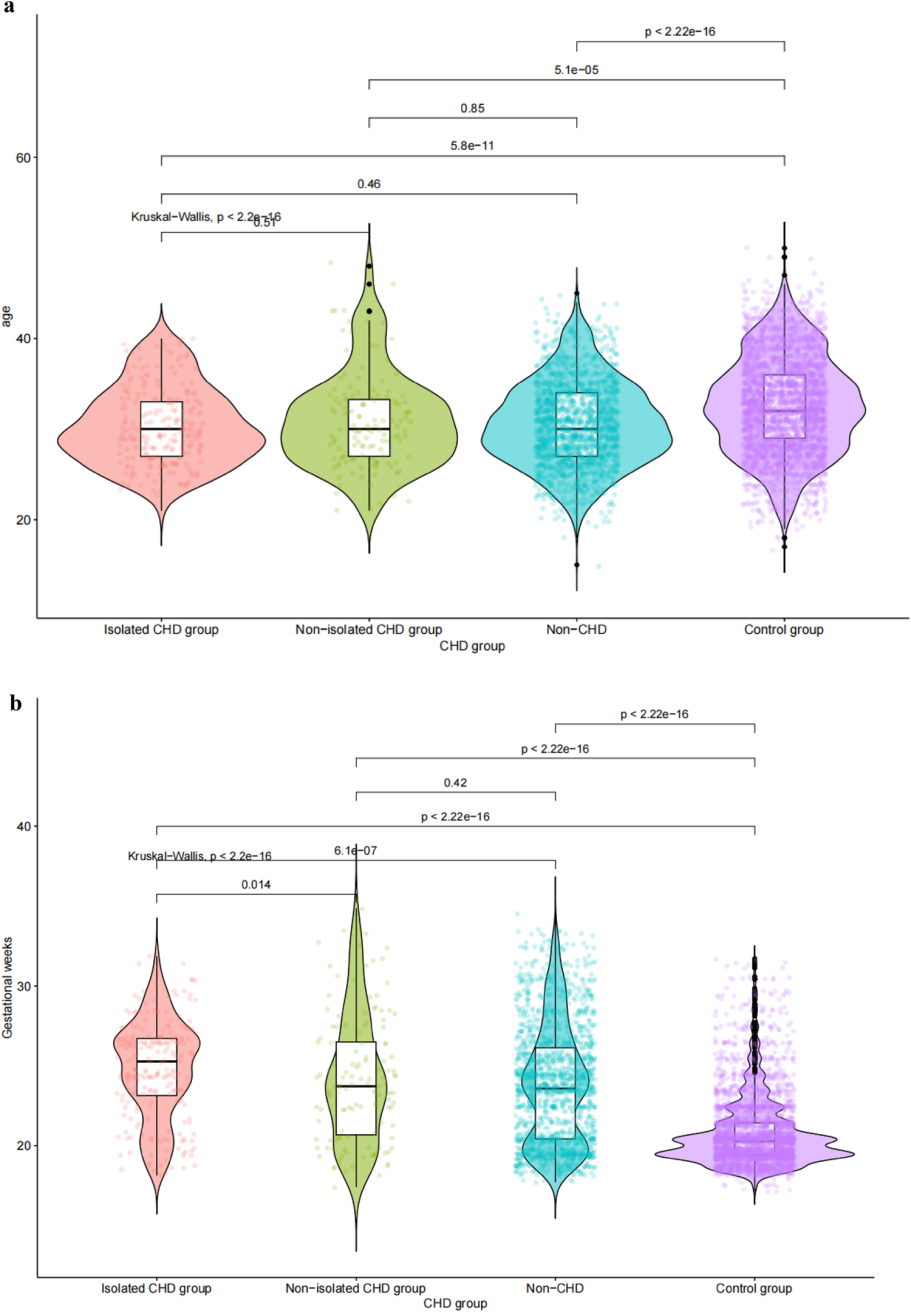
a The ages and gestational weeks of the four groups of pregnant women. b The ages and gestational weeks of the four groups of pregnant women.

This section describes the sociodemographic characteristics of the 5,116 pregnant women, including age, gestational age, and pregnancy, and compares these characteristics between the isolated CHD, non-isolated CHD, non-CHD, and normal groups. The results showed that all variables were significantly different among the four groups. Most of the women were between 30 and 40 years old, and the mean age of the normal group (32 years) was significantly higher than that of the other group (32 years) (F = 189.55, *P* < 0.001) (Fig. 2a). There were also significant differences in gestational weeks among the four groups. The gestational weeks of the control group were significantly shorter than those of the other groups(Fig. 2b).

In the isolated CHD group, the proportion of women with one pregnancy (39.24%) and three or more pregnancies (36.29%) was higher, while in the non-isolated CHD group, the proportions of one (33.09%), two (34.56%), and three or more (32.35%) pregnancies were similar. Notably, in the normal group, over half (52.30%) of pregnant women had one birth experience, which was significantly higher than in the other groups (Fisher = 110.01, *P* < 0.001). High-risk factors associated with ultrasound abnormalities (isolated CHD, non-isolated CHD, and non-CHD) included advanced maternal age (17–19%), adverse pregnancy history, high risk of non-invasive DNA (4–15%), and chromosomal abnormalities in one couple (0%–6%). Each of these risk factors was significantly different among the four groups (*P* < 0.001) (Table 1).

**Table 1:** Basic information of pregnant women in the four groups(%).

Variable	Isolated CHD group	Non-isolated CHD group	Non-CHD group	Normal group	χ^2^/Fisher	*P*-Vale
n	237	136	1632	3111		
Number of pregnancies						
1	93(39.24)	45(33.09)	564(34.56)	674(21.66)	131.61	< 0.001
2	58(24.47)	47(34.56)	477(29.23)	922(29.64)		
≥ 3	86(36.29)	44(32.35)	591(36.21)	1515(48.70)		
Number of births						
0	133(56.12)	68(50.00)	791(48.47)	1107(35.58)	110.01	< 0.001
1	93(39.24)	55(40.44)	711(43.57)	1627(52.30)		
2	10(4.22)	11(8.09)	119(7.29)	341(10.96)		
≥ 3	1(0.42)	2(1.47)	11(0.67)	36(1.16)		
High risk factor						
Yes	75(31.65)	44(32.35)	417(25.55)	1996(64.16)	688.108	< 0.001
No	162(68.35)	92(67.65)	1215(74.45)	1115(35.84)		
Types of risk factors						
Advanced age	43(18.14)	24(17.65)	324(19.85)	1119(35.97)	159.47	< 0.001
History of adverse pregnancy	37(15.61)	18(13.24)	76(4.66)	563(18.10)	165.81	< 0.001
Noninvasive DNA is high risk	7(2.95)	7(5.15)	63(3.86)	667(21.44)	306.28	< 0.001
One of the couples has a chromosomal abnormality	2(0.84)	0(0.00)	6(0.37)	54(1.74)	19.87	< 0.001

Variable	Isolated CHD group	Non-isolated CHD group	Normal group	χ^2^/Fisher	*P*-Vale
N	237	136	3111		
Aneuploidies	9(3.80)	23(16.91)	240(7.71)	20.99	< 0.001
pathogenic CNVs	5(2.11)	5(3.68)	83(2.67)	0.93	0.60
22q11.2	5(2.11)	1(0.74)	11(0.35)	16.961	< 0.001
Others	0(0.00)	4(2.94)	72(2.31)		
likely pathogenic CNVs	0(0.0)	0(0.0)	10(0.32)	0.037	1.00
pathogenic and likely pathogenic CNVs	14(5.91)	28(20.59)	411(13.21)	17.59	< 0.001
variation unknown significance CNVs	9(3.80)	6(4.41)	181(5.82)	2.09	0.35

### Distribution of aneuploidies and CNVs across groups

**Fig. 3:**
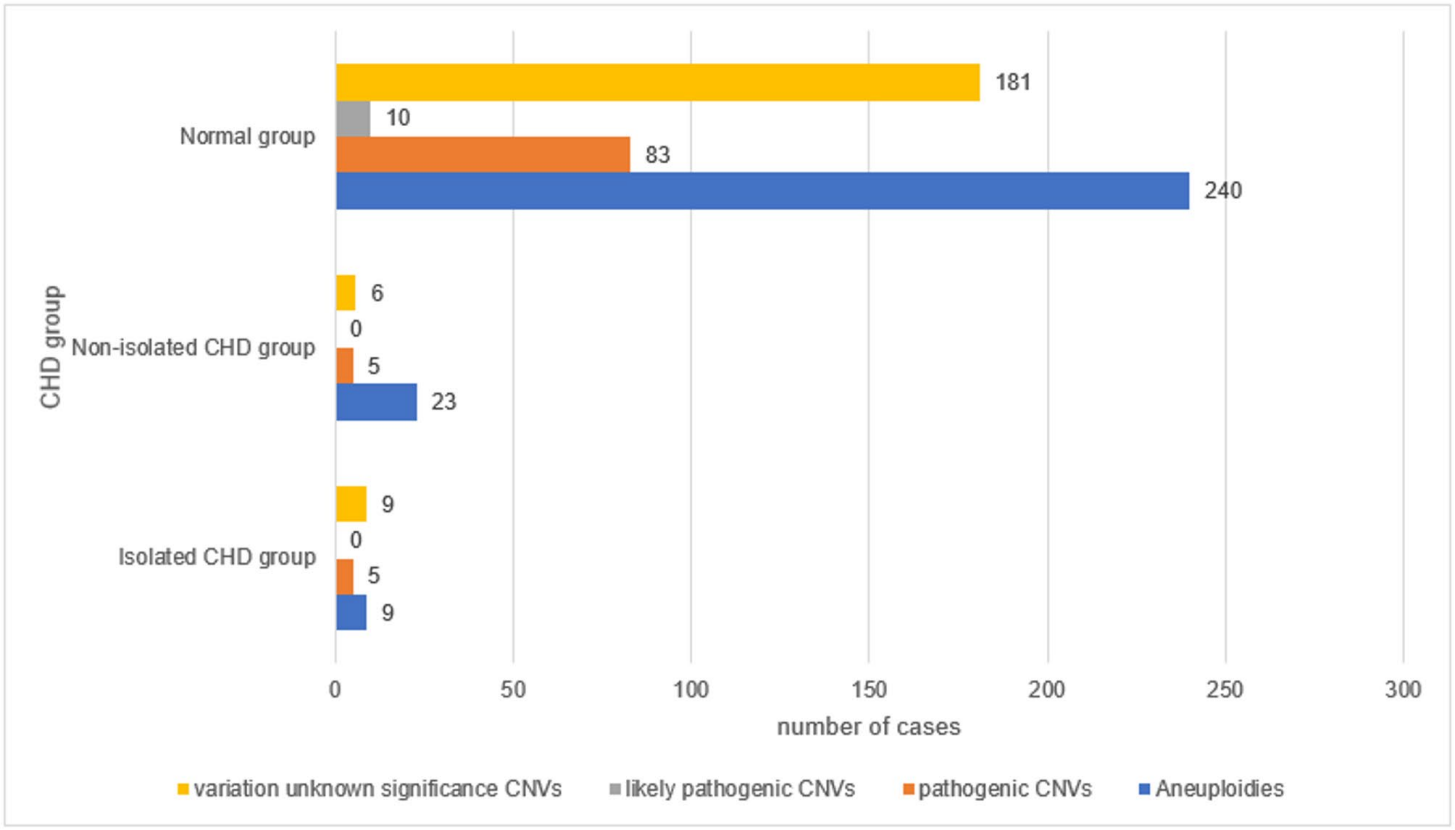
Incidence of aneuploidies, pathogenicity, likely pathogenicity, and variant CNVs of uncertain significance in four groups of pregnant women (%).

Based on chromosome karyotype results and SNP-based CMA analysis, we categorized the test results into aneuploidies, pathogenic CNVs, LP CNVs, and CNVs of uncertain significance. Aneuploidies were the most common abnormality (376 cases, 7.35%), while pathogenic CNVs (139, 2.72%) occurred about eight times as frequently as LP CNVs (16, 0.31%) and CNVs of uncertain significance (290, 5.67%). The non-isolated CHD group had the highest proportion of aneuploidies (16.91%), which was about five times greater than that of the isolated CHD group (3.8%). The proportions of pathogenic CNVs in the three groups was similar (ranging from 2.11% to 3.68%), with the variation rate of 22q11.2 in the normal group (11 cases, 0.35%) being significantly lower than that of the non-isolated CHD group (one case, 0.74%). The incidence of CNVs of uncertain significance in each group showed minimal variation between 3% and 5% (Fig. 3).

### Distribution of aneuploidies across groups

The results showed significant differences in the distribution of CNVs among the three groups of pregnant women. The detection rates of trisomy 21 (χ^2^=12.5, *P* = 0.002), trisomy 18 (Fisher = 23.00, *P* < 0.001), and “other aneuploidies (which include rare autosomal trisomies, sex chromosomal aneuploidies, etc.)” (Fisher = 1.27, *P* < 0.051) were statistically different between the groups. The proportion of Trisomy 21 was highest in the non-isolated CHD group (12/136, 8.82%), which was significantly greater than in the isolated CHD (3/237, 1.27%; *P* < 0.001), and normal groups (111/3,111, 3.57%). Similarly, the detection rate of trisomy 18 was much higher in the non-isolated CHD group (8/136, 5.88%) than in the isolated CHD group (0.42%), normal group (0.55%). The incidence of “other aneuploidies” was highest in the normal group (107/3,111, 3.44%), followed by the non-isolated CHD group (3/136, 2.21%), and the isolated CHD group (5/237, 2.11%),. Trisomy 13 was extremely rare in all groups (0.00% to 0.10%), showing no significant group differences (Table 2).

**Table 2:** Distribution of aneuploidies in the four groups of pregnant women(%).

Variable	Isolated CHD group	Non-isolated CHD group	Normal group	χ^2^/Fisher	*P*-Vale
n	237	136	3111		
Trisomy 21	3(1.27)	12(8.82)	111(3.57)	12.50	0.002
Trisomy 18	1(0.42)	8(5.88)	17(0.55)	23.00	< 0.001
Trisomy 13	0(0.00)	0(0.00)	3(0.10)	0.853	1.00
Other aneuploidies	5(2.11)	3(2.21)	107(3.44)	1.27	0.51

### Comparison of fragment sizes of different CNVs across groups

While small CNVs (< 1 Mb) (e.g., GATA4, 22q11.2) are linked to CHD (e.g., VSD, conotruncal defects), their low penetrance necessitates additional genetic (e.g., NKX2-5 SNVs) or environmental (e.g., maternal diabetes) factors to cause CHD. Larger CNVs (5 Mb) are more significantly associated with severe congenital diseases, mental retardation, developmental delay, and other diseases, rendering them more likely to be identified and valued in clinical diagnosis^15^. We collated and analyzed all CNV fragments with chromosomal variation, identifying 234 individuals with such variations. The sizes of these variations were classified into three groups: <1 Mb, 1–5 Mb, and > 5 Mb. No significant differences were observed in the distribution of CNV fragment sizes across the four groups. In the isolated CHD group, fragments measuring 1–5 Mb were the most prevalent, accounting for 2.95%, approximately twice the proportion of fragments ≥ 5 Mb (1.47%). Meanwhile, the normal group exhibited the lowest proportion of small fragments (< 1 Mb) at 0.9%, with its highest proportion observed in the 1–5 Mb range at 2.6% (Table 3).

**Table 3:** Compares the distribution frequency of CNV fragment size in the four groups of pregnant women (%).

Variable	Isolated CHD group	Non-isolated CHD group	Normal group	Fisher	*P*-Vale
n	237	136	3111		
<1 Mb	1(0.42)	1(0.74)	28(0.90)	0.24	0.90
1–5 Mb	7(2.95)	4(2.94)	80(2.60)	0.47	0.80
≥ 5 Mb	0(0.00)	2(1.47)	30(0.96)	2.82	0.20

### Specific distribution of pathogenic CNVs across groups

The distribution of pathogenic CNVs among the four groups revealed significant genomic heterogeneity and site-specific enrichment patterns. Notably, CNVs in regions such as 15q11.2, 16p11.2, 22q11.21, and Xp 22.31 were recurrent in multiple groups, with their losses and gains contributing to phenotypic variability. Among them, the 22q11.21 region showed prominent group differences, with the isolated CHD group displaying larger deletions (e.g., losses (22q11.21) (3.15–3.1 Mb)), while the normal group had smaller losses (1.08–2.80 Mb). The 15q11.2 locus showed complex variations (e.g., UBE3A, SNRPN, which are critical for cardiac development-related pathways) that are essential for elucidating genotype-phenotype correlations in CHD. The non-isolated CHD group mainly exhibited eight losses of varying sizes (0.312–0.855 Mb), while the normal group had seven smaller losses (0.436–0.548 Mb).

Similarly, the fragment length of the 16p11.2 losses showed group differences. The CHD group had losses measuring 0.761 Mb, The normal group had losses measuring 0.303 Mb. In sex chromosome CNVs, Xp 22.31 emerged as a key region, and its losses (1.06–1.69 Mb) were prevalent across all groups. However, the normal group exhibited larger loss fragments (1.46–1.69 Mb), often combined with Y chromosome losses, such as losses (Yq11.221q11.23) (9.146 Mb). These findings indicate that while the normal group did not present with overt congenital abnormalities, they harbored more substantial chromosomal deletions compared to other study groups, with a distinct predilection for losses in specific Y chromosome regions. There were multiple cases of whole-arm abnormalities, such as gains (9p24.3p13.1) (38.45–38.56 Mb) and losses (18q21.33q23) (17.09–18.88 Mb), suggesting a broader effect on systemic development. Additionally, the normal group was enriched at the 17p12 site CNVs (1.342–1.485 Mb) and Xp 21.1 losses (0.135–0.364 Mb) (Table 4).

**Table 4:** The distribution of pathogenic CNV in the four groups.

Isolated CHD group	Non-isolated CHD group	Normal group
		losses(1q21.1q21.2)(2.045 Mb)
		losses(1q23.3q25.1)(9.85 Mb)
		losses(2p16.3)(0.293 Mb)
		losses(2p16.3)(0.399 Mb)
		losses(2p16.3)(0.433 Mb)
		gains(3p14.1)(1.24 Mb)
		gains(4p16.3p16.1)(8.65 Mb)
		losses(5p13.2)(0.47 Mb)
		losses(6q27)(5.022 Mb)
	losses(7q11.23)(1.49 Mb)	losses(7q11.23)(1.486 Mb)
		gains(7q11.23)(3.61 Mb)
	gains(7q34q36.3)(17.43 Mb)	
		losses(7q36.1q36.3)(9.9 Mb)
	gains(9p24.3p13.1)(38.45 Mb)	gains(9p24.3p13.1)(38.56 Mb)
		gains(10p15.3)(1.83 Mb)
		gains(10q21.1)(1.57 Mb)
		losses(11q22.1q22.3)(9.56 Mb)
		gains(12p13.33p12.2)(19.891 Mb)
		gains(13q11q13.3)(16.8 Mb)
		gains(13q14.13q34)(69.20 Mb)
		gains(13q21.33q34)(41.5 Mb)
		losses(15q11.2)(0.436 Mb)
		losses(15q11.2)(0.444 Mb)
		losses(15q11.2)(0.506 Mb)
		losses(15q11.2)(0.507 Mb)
		losses(15q11.2)(0.507 Mb)
		losses(15q11.2)(0.512 Mb)
		losses(15q11.2)(0.512 Mb)
		losses(15q11.2)(0.548 Mb)
		losses(15q11.2)(0.845 Mb)
		gains(15q11.2q13.1)(5.26 Mb)
		gains(15q11.2q13.1)(5.64 Mb)
	losses(16p11.2)(0.761 Mb)	losses(16p11.2)(0.303 Mb)
		losses(16p11.2)(1.01 Mb)
		gains(16p11.2)(0.586 Mb)
		gains(16p11.2)(0.609 Mb)
		gains(16p11.2)(0.758 Mb)
		losses(16p13.12p13.11)(1.77 Mb)
		losses(17p12)(1.39 Mb)
		losses(17p12)(1.411 Mb)
		losses(17p12)(1.418 Mb)
		gains(17p12)(1.342 Mb)
		gains(17p12)(1.40 Mb)
		losses(17q12)(1.42 Mb)
		losses(17q12)(1.485 Mb)
		losses(18p11.32p11.31)(4.843 Mb)
		losses(18q21.33q23)(17.09 Mb)
losses(22q11.21)(3.152 Mb)	losses(22q11.21)(3.169 Mb)	losses(22q11.21)(1.08 Mb)
losses(22q11.21)(3.15 Mb)		losses(22q11.21)(2.80 Mb)
losses(22q11.21)(3.1 Mb)		
losses(22q11.21)(1.81 Mb)		
losses(22q11.21)(3.15 Mb)		
		gains(22q11.21)(2.49 Mb)
		gains(22q11.21)(2.49 Mb)
		gains(22q11.21)(2.812 Mb)
		gains(22q11.21)(2.881 Mb)
		gains(22q11.21)(2.89 Mb)
		gains(22q11.21)(3.008 Mb)
		gains(22q11.21)(3.15 Mb)
		losses(22q11.21q11.22)(1.16 Mb)
		gains(Xp11.21q28)(99.74 Mb)
		losses(Xp11.22q28)(99.05 Mb)
		losses(Xp11.22q28)(99.66 Mb)
		losses(Xp21.1)(0.135 Mb)
		losses(Xp21.1)(0.364 Mb)
		losses(Xp22.12p21.1)(16.85 Mb)
		losses(Xp22.12p21.2)(11.481 Mb)
		losses(Xp22.31)(1.06 Mb)
		losses(Xp22.31)(1.46 Mb)
		losses(Xp22.31)(1.68 Mb)
		losses(Xp22.31)(1.69 Mb)
		losses(Xp22.31)(1.69 Mb)
		losses(Xp22.31)(1.69 Mb)
		losses(Xp22.33p11.1)(58.36 Mb)
		losses(Xp22.33p22.32)(5.2 Mb)
		gains(Xq28)(0.516 Mb)
		losses(Yq11.221q11.23)(9.146 Mb)
		losses(Yq11.221q11.23)(9.22 Mb)
		losses(Yq11.223q11.23)(3.6 Mb)
		gains(Yq11.221q11.23)(13.39 Mb)

### Specific distribution of potentially pathogenic CNVs across groups

Table 5 details the distribution of potentially pathogenic CNVs among the four groups. The non-isolated CHD group had the highest number and greatest diversity of CNVs, including large segment gains such as gains (2q35q37.3) (25.96 Mb). This region covers multiple development-related genes (e.g., HOXD cluster) and may be associated with non-cardiac phenotypes (e.g., skeletal malformation). In addition, losses (6q27) (5.02 Mb) were observed in both the non-isolated CHD and normal groups, with highly similar fragment lengths (5.02 Mb vs. 5.022 Mb). Partly overlapping CNVs were observed in normal groups. Sex chromosome-related CNVs, such as losses (Xp21.2p21.1) (0.113 Mb) and losses (Yq11.223q23) (3.6 Mb), were exclusively found in the normal groups.

**Table 5:** The distribution of likely pathogenic CNV in the four groups.

Isolated CHD group	Non-isolated CHD group	Normal group
		losses(2p16.3)(0.293 Mb)
		losses(5p13.2)(0.47 Mb)
		losses(6q27)(5.022 Mb)
		gains(16p11.2)(0.609 Mb)
		losses(Xp21.1)(0.364 Mb)
		losses(Yq11.223q11.23)(3.6 Mb)

### Specific distribution of uncertain significance (variation unknown significance, VUS)

Table 6 summarizes the distribution of VUS among the four groups. The number of VUS was significantly higher in the normal groups than in the CHD group. CNVs in the 22q11.21 region showed a complex distribution, with losses such as losses (22q11.21q11.22) (1.16 Mb) being predominantly found in the isolated CHD group, whereas gains such as gains (22q11.21)(1.08–1.5 Mb) had enriched repeats in both the normal groups. Repeated losses in the 15q13 region (3.18 Mb) and gains in 16p11.2 (0.61 Mb) were observed in the non-isolated CHD group, partially overlapping with Tables 4 and 5. Sex chromosome-associated VUS (e.g., Xp 22.31 gains and Y chromosome large losses) were enriched in the normal groups, including gains (Xp22.31) (1.68–2.4 Mb) and losses (Yp11.31q11.221) (16.28 Mb). Notably, the isolated CHD group demonstrated a low number of VUS, with no significant clustered variants identified. Widespread VUS, such as gains (8p23.2) (2.25 Mb) and losses (13q14.3q21.2) (6.66 Mb) in the normal group, may be associated with population polymorphisms.

**Table 6:** The distribution of variation unknown significance CNV in the four groups.

Isolated CHD group	Non-isolated CHD group	Normal group
		losses(1q44)(2.46 Mb)
		gains(2p12)(1.18 Mb)
		gains(2p16.3p16.2)(2.70 Mb)
		gains(2p22.1)(1.56 Mb)
		gains(3p12.3)(1.06 Mb)
		gains(3p14.1)(1.24 Mb)
		gains(3p26.3)(2.49 Mb)
		losses(4p13p12)(4 Mb)
		losses(4q22.1)(0.551 Mb)
		gains(4q34.3)(1.72 Mb)
		gains(4q34.3)(1.73 Mb)
		gains(4q34.3)(1.76 Mb)
		losses(4q35.1q35.2)(4.11 Mb)
losses(5p13.3p13.2)(1.96 Mb)		
		losses(6p12.3)(1.01 Mb)
		gains(6p25.1p24.3)(2.58 Mb)
		losses(6q15q16.1)(9.88 Mb)
		gains(6q21q22.1)(8.414 Mb)
		gains(8p21.2p21.1)(1.53 Mb)
		gains(8p23.2)(2.25 Mb)
		gains(8q24.23q24.3)(1.14 Mb)
		losses(9q31.3)(0.654 Mb)
		losses(10p11.21p11.1)(1.10 Mb)
		gains(10q11.22q11.23)(1.315 Mb)
	losses(10q21.1)(1.36 Mb)	
		losses(10q22.3)(0.511 Mb)
		gains(11p12)(1.78 Mb)
		gains(11q14.3)(1.46 Mb)
		gains(12q11q12)(1.14 Mb)
		losses(13q12.12)(1.3 Mb)
		losses(13q12.12)(1.42 Mb)
		losses(13q14.2q14.3)(4.17 Mb)
		losses(13q14.3q21.2)(6.66 Mb)
		losses(14q22.1q22.2)(1.23 Mb)
		gains(14q22.2q22.3)(1.40 Mb)
		losses(14q24.3q31.3)(8.369 Mb)
	losses(15q13.1q13.2)(2.28 Mb)	
		gains(15q13.1q13.2)(1.08 Mb)
		losses(15q25.2q25.3)(0.88 Mb)
		gains(16p11.2)(0.61 Mb)
		losses(16p12.2)(0.702 Mb)
		gains(16p13.11)(1.224 Mb)
		gains(16p13.11)(1.588 Mb)
		gains(16p13.11p12.3)(2.74 Mb)
		gains(16q23.1)(1.31 Mb)
		gains(17q11.1q11.2)(1.72 Mb)
		gains(18p11.31p11.23)(3.53 Mb)
		losses(18q23)(1.416 Mb)
		gains(19q13.41q13.43)(6.799 Mb)
		gains(20p12.2p12.1)(4.672 Mb)
		gains(21q21.1q21.2)(5.015 Mb)
		gains(21q22.3)(1.25 Mb)
		losses(22q11.1q11.21)(1.45 Mb)
losses(22q11.21q11.22)(1.16 Mb)		
losses(22q11.22q11.23)(0.655 Mb)		
		gains(22q11.21)(1.08 Mb)
		gains(22q11.21)(1.5 Mb)
		gains(22q11.22q11.23)(2.01 Mb)
		gains(22q11.23)(1.306 Mb)
		gains(22q11.23)(1.31 Mb)
		losses(22q13.31)(1.02 Mb)
		gains(Xp21.1)(0.078 Mb)
		gains(Xp21.1)(0.337 Mb)
		gains(Xp22.31)(2.4 Mb)
		gains(Xp22.33)(1.51 Mb)
		gains(Xq21.32q22.1)(7.19 Mb)
		losses(Yp11.31q11.221)(16.28 Mb)

## Comment

CHD has emerged as a major social and public health problem, compromising the health of children and the overall population.Therefore, prenatal screening for CHD is particularly critical, and ultrasonography serves as an essential component of this screening process^17^.Prenatal ultrasound examination allows for timely detection of fetal malformations and abnormalities, and further chromosome examination is needed for fetuses with abnormal results. This study used SNP-based CMA technology to detect fetuses with cardiac abnormalities noted during ultrasound evaluations, yielding a series of meaningful results. As a high-resolution genetic detection technology, SNP-based CMA can detect genome-wide chromosomal CNV, providing a new perspective on the etiological diagnosis of fetal ultrasound-detected cardiac abnormalities.

Several studies have shown that maternal age of 35 years (advanced age) is a risk factor for CHD^18^. A study based on propensity score matching found a positive association between maternal age and the risk of CHD (odds ratio (OR) = 1.013, 95% confidence interval (CI): 1.002, 1.024)^19^. However, the present study found that the proportion of women with advanced-age in the normal group was 35.97%, significantly higher than 17%–−19% observed in the CHD group, potentially attributed to sample selection bias^20^.

The main risk factors in the group with abnormal ultrasound included adverse pregnancy history and high risk of non-invasive DNA. Some studies have highlighted these factors as significant risk determinants for abnormal fetal development^21^, aligning with the findings of this study. In this study, among the classification of risk factor in the normal group, the proportions of “advanced age” (35.97%) and “Non-invasive DNA is high risk” (21.44%) were relatively high, possibly due to the overall older age of pregnant women in this group and the prenatal screening strategy employed. This finding further confirms that adverse pregnancy history is the main risk factor for fetal abnormalities.

CNVs are key risk factors for CHD. Over the past decades, several CNVs have been associated with CHD, including tetralogy, pulmonary atresia, and VSDs^22^. To date, more than 55 human genes have been linked to CHD^23^, and more will be identified with advancements in whole-genome sequencing. This study showed that aneuploidy variation were the most common, especially in the non-isolated CHD group, which was significantly higher compared to the other groups. This is consistent with numerous national and international studies^24^. Some studies highlighted aneuploidies (such as trisomy) as the main type of chromosomal abnormality^25^, leading to congenital malformations and abortions. The proportion of aneuploidies in the non-isolated CHD group was five times greater than in the isolated CHD group, suggesting that abnormalities chromosome number may lead to more complex phenotypes by affecting multiphylogeny. This finding is also consistent with the strong association between chromosomal number abnormalities and complex congenital malformations described in large cohort studies, such as the DECIPHER database.The results of this study showed that the proportion of abnormal CNVs in the CHD group was significantly lower than that of non-isolated CHD., consistent with the findings by Fenglei Ye et al., which reported a total detection rate of pathogenic CNVs in the CHD group (8.2%) that was significantly lower than that in the non-isolated CHD group (14.7%)^26^.

The results of this study showed that the detection rate of trisomy 21 in the non-isolated CHD group (8.82%) was significantly higher than that of the other groups (1.27–4.47%), suggesting that it was closely related to the occurrence of complex cardiac malformations. Trisomy 21 (Down syndrome) is an aneuploid condition known to be highly associated with CHD, affecting about 40–50% of children with CHD (e.g., VSD). A cohort study found that the rate of chromosomal abnormalities was significantly higher in the non-isolated CHD group (31.8%) compared to the isolated CHD group (23.6%), with trisomy 21 as the main type^27^. These findings support the high incidence of trisomy 21 in the non-isolated CHD group in the present study. The proportion of trisomy 21 with CHD in European and American populations (approximately 45%) slightly higher than those in the Asian population (approximately 35%), which may be due to differences in genetic background or prenatal diagnostic strategies^28^. In this study, the detection rate of trisomy 18 in the non-isolated CHD group (5.88%) was considerably higher than the rates in the other groups (0.42%%–1.04%).

Trisomy 18 (Edwards syndrome) is often associated with multiple systemic malformations, and its cardiac malformations (such as VSD and TOF) are mostly complex. One study analyzed chromosomal abnormalities in 179 CHD fetuses and found that the incidence of CHD in trisomy 18 at 73.7% (14/19), with most cases characterized as “complex cardiac malformations,” including large VSD and TOF. In contrast, trisomy 21 had a lower proportion of non-isolated CHD, mainly presenting as endocardial cushion defects^24^, consistent with the findings of this study.

A prenatal study based on CMA in 2022 showed that the detection rate of trisomy 18 was 4.8% in the non-isolated CHD group, which is similar to the results of this study (5.88%), suggesting that small differences may be due to differences in technical sensitivity (Probe density, sample quality (such as placental chimerism, maternal blood contamination) and data analysis standards). The results of this study showed that trisomy 13 was extremely rare in all groups (0.00–0.10%). The live birth rate of trisomy 13 (Patau syndrome) is considerably low (about 1/10,000)^29^, and more than 95% of embryos are naturally eliminated in early pregnancy. This is consistent with the birth defect monitoring data in many countries worldwide, such as the United States and Japan.

Chromosomal microdeletions and microduplications, such as 22q11.2, microdeletion syndrome and Williams-Campbell syndrome, are associated with fetal CHD^30^. The most common pathogenic CNVs in this study included 22q11.2 losses, which is consistent with the existing literature. Multiple studies have found an association between conic artery trunk malformation (conotruncal defects) and 22q11.2 losses syndrome^31^.

The findings of this study further contribute to research on the relationship between CNV segment size distribution and CHD. Despite the absence of significant differences between groups, the size distribution of CNV fragments across different groups displayed notable characteristics. For instance, the proportion of CNVs ranging from 1 to 5 Mb was higher across the four groups, indicating that while no overall differences were detected, the underlying genetic characteristics of the different groups may still demonstrate slight discrepancies, providing insights for further research into the role of CNVs in the development of CHD.

In addition, this study suggests that various factors should be considered when analyzing the relationship between CNVs and disease, including the diversity of samples and the accuracy of detection techniques, to elucidate their internal relationships more comprehensively and accurately. Similar to this study, some international studies found no significant differences in CNV fragment size distributions across specific diseases and normal groups^32^, which may be related to the multifactorial nature of diseases and the complexities associated with samples encompassing varied sample types (e.g., prenatal amniotic fluid, maternal plasma cfDNA), chromosomal mosaicism (different abnormal cell proportions), differential maternal cell contamination (key in prenatal samples), and clinical heterogeneity of participants (e.g., comorbidities). This study suggests that various factors should be considered when analyzing the association between CNVs and disease—including sample diversity and the accuracy of detection techniques—to more comprehensively and accurately elucidate the true relationship between these two entities.

This study showed a diverse range of pathogenic CNV types and their distributions. Numerous studies have shown that CNV in specific chromosomal regions is closely associated with CHD and that losses or gains in the 22q11.2 region are CNV types strongly linked to CHD^33^. In this study, variants with different copy numbers and interval ranges were identified in the 22q11.21 region across the isolated CHD, and normal groups. In the isolated CHD group, five losses at 22q11.21, with variant fragment sizes spanning from 1.81 Mb to 3.15 Mb. This finding is consistent with the results of international studies showing variations in this region are associated with CHD, which further confirms the region’s role in the pathogenesis of CHD.

In this study, the 16p11.2 region showed multiple variation types and copy number changes in non-isolated CHD and normal groups, indicating that genetic variations in this region are prevalent among different types of CHD and normal control populations.

Numerous studies have also focused on constructing CHD profiles correlated with CNVs and found CNVs associated with CHD in multiple chromosomal regions. In this study, the 1q21.1q21.2 region showed multiple CNVs in the normal groups, suggesting that variations in this region may also play an important role in the pathogenesis of CHD in China. Some studies compared the differences in CNV across different groups and found differences in CNV distributions in specific chromosomal regions in the CHD group compared with the normal group^34^. This study documented clear differences in CNV types and copy numbers in each chromosomal region across different groups. For example, there existed differences in variations in the 15q11.2 region the normal groups. These results provide basic data for subsequent in-depth analysis of the differences between groups.

The distribution of LP CNV in the isolated CHD, non-isolated CHD, and normal groups was analyzed.Existing literature has established strong correlations between CNVs in specific chromosomal regions and CHD. For example, CNV variants in the 15q11.2 region are widely reported to be associated with CHD^35^, consistent with the findings of this study.

Our results showed that the aneuploidy rate in non-isolated CHD fetuses was 16.91%, which was approximately five times higher than that in isolated CHD (3.8%, *P* < 0.001), with trisomy 21 (8.82%) and trisomy 18 (5.88%) being the most common. This finding aligns with previous studies^36,37^ that reported higher chromosomal aberration rates in non-isolated CHD, as non-isolated CHD often indicates systemic developmental abnormalities associated with chromosomal aneuploidies.

From a clinical perspective, this highlights the critical utility of SNP-based CMA in non-isolated CHD.

In contrast to aneuploidies, the aneuploidy rate in isolated CHD was relatively low (3.8%). However, SNP-based CMA still showed important clinical value in this subgroup:

We identified five cases of 22q11.2 deletions in the isolated CHD group—22q11.2 deletion syndrome is a major genetic cause of isolated CHD (especially conotruncal defects), and these deletions may be missed by conventional karyotyping due to their small size^38,39^. SNP-based CMA, with its high resolution, can effectively detect such pathogenic CNVs, thereby supplementing the etiological diagnosis of isolated CHD.

### Study limitations and strengths

This study has some limitations, including a lack of data on follow-up of pregnant women undergoing amniocentesis and maternal and infant outcomes. This makes it difficult to comprehensively evaluate the association between SNP chip analysis results, development of fetal CHD, maternal pregnancy status, and maternal and infant health status after delivery. Consequently, this limitation restricts the applicability of research findings in clinical decision-making.

Nevertheless, our study has many strengths, including being the first large-scale SNP data analysis undertaken in Xinjiang, which considers differences in regional and population genetic backgrounds, providing a unique perspective on the etiology of fetal CHD. In addition, CHD cases were carefully categorized into distinct groups, and detailed information on all pathogenic, LP, and clinically significant is comprehensively presented. This comprehensive approach allows for extensive analysis of the genetic mechanism of different CHD and lays a solid foundation for precise diagnosis and genetic consultation in future investigations.

### Future research

Future efforts will involve establishing a large-scale database of fetuses with cardiac abnormalities noted on ultrasound examinations, integrating genetic information, ultrasound images, clinical data, and other data, especially maternal and infant outcome data from follow-up studies. By leveraging big data analysis and artificial intelligence technologies, potential disease markers and diagnostic models can be mined to improve early diagnosis and precision treatment approaches of fetal ultrasound-detected cardiac abnormalities.

## Conclusions

The findings of this study suggest that enhancing the positive rate of etiological diagnosis for cases of fetal ultrasound-detected cardiac abnormalities is recommended. In clinical practice, for fetuses with cardiac structural abnormalities but normal chromosome karyotyping results, SNP chip analysis can be used as an effective complementary testing method to provide more accurate information for clinical diagnosis and genetic counseling. Understanding the genetic etiology of fetuses with cardiac abnormalities noted on ultrasound can facilitate more precise genetic counseling. For families with pathogenic genetic variants, physicians can provide more detailed genetic risk assessment and fertility advice to help them make informed decisions. Furthermore, for certain genetically predisposed heart diseases, early genetic diagnosis can support risk assessment of family members, enabling the implementation of early prevention and intervention against the disease.

## Supplementary Information

Below is the link to the electronic supplementary material.

Supplementary Material 1

## Data Availability

The datasets used and/or analyzed during the current study are available from the corresponding author upon reasonable request.
